# The Genetic Polymorphisms of DNA Repair Gene *RAD51* and Their Role as a Predictive Biomarker for Breast Cancer Risk and Response to Radiotherapy in Iraqi Women

**DOI:** 10.31557/APJCP.2026.27.1.345

**Published:** 2026-01-22

**Authors:** Haael Subhi Abbas, Ali Abdul Hussein Mahdi, Nihad Khalawe Tektook

**Affiliations:** *Department of Techniques for Pathological Analysis, College of Health and Medical Techniques, Middle Technical University, Baghdad, Iraq.*

**Keywords:** RAD51, breast cancer, polymorphisms, radiation therapy, biomarker

## Abstract

**Objective:**

Variation in the DNA repair gene *RAD51* has been linked to breast cancer (BC), the most common cancer worldwide, raising concerns about radiotherapy (RT) resistance. This study aims to determine *RAD51* levels and polymorphisms in Iraqi women with BC before and after RT and to evaluate their potential as cancer risk and treatment response biomarkers.

**Methods:**

This follow-up study examined 90 blood samples from 30 newly diagnosed BC patients aged 34–56 years before and after RT at the Al-Amal National Hospital for Cancer Management in Baghdad collected from July 2024 to January 2025, and from 30 age- and sex-matched healthy controls. In ELISA, serum *RAD51* levels were estimated. *RAD51* SNPs were genotyped through PCR amplification with specific primers and DNA sequencing

**Result:**

Post-RT, BC patients had significantly more *RAD51* levels than pre-treatment patients and controls (2.51±0.70 vs. 1.22±0.19 vs. 1.27±0.21 ng/ml). The Iraqi population’s rs1801320 (G>C) and rs1801321 (G>T) SNPs in exon 1 of the *RAD51* were identified and recently registered at the NCBI (PV661827 and PV661828). The *RAD51* (G>T) polymorphism’s GT genotype and T allele were significant risk factors for BC (OR=11.07, CI=3.20-37.87 and OR=4.92, CI=1.82-13.35). BC patients had a lower variation post-RT compared to prior (20.0% vs. 27.0%). The *RAD51* (G>C) polymorphism GC genotype or C allele was not linked with BC risk (OR=2.25, CI=0.50-9.93 and OR=2.11, CI=0.503-8.87, P>0.05).

**Conclusion:**

The Iraqi BC risk was associated with the *RAD51* (G>T) polymorphism. GT genotype / T allele patients responded well to RT, indicating that *RAD51* might predict cancer risk and RT effectiveness. Iraqi women may benefit from the *RAD51* (G>C) GG genotype in protection against BC.

## Introduction

Breast cancer (BC) encompasses a range of diseases characterized by the uncontrolled division and alteration of cells within breast tissue, leading to tumour formation [1]. Breast cancer continues to be a primary contributor to cancer-related deaths among women, highlighting significant disease diversity, metastasis, and treatment resistance [2]. Consequently, early recognition correlates with decreased mortality rates [3]. In Iraq, BC is recognized as the most common malignancy, accounting for 21% of cases [4]. Environmental, genetic, and lifestyle factors may contribute to the emergence of BC. About 30% of BC cases may be affected by modifiable factors [5].

Radiotherapy (RT) is a localized treatment that employs high-energy photons or particles to eliminate cancerous cells [6]. Adjuvant RT is frequently administered post-mastectomy or lumpectomy to reduce the risk of cancer recurrence [7]. The reactions of cancer patients to RT can differ significantly, reflecting the fundamental systems governing the response to radiation damage [8]. Radioresistance constitutes a major obstacle to enhancing treatment outcomes, resulting in RT failing, persistent tumors, and poor prognosis [9]. It can arise from various mechanisms such as tumor heterogeneity, the surrounding microenvironment, and multiple genetic modifications. The presence of the DNA-dependent recombinase *RAD51* represents a notable alteration [10].

The *RAD51* gene, situated on the long arm of chromosome 15, comprises 14 exons that encode the DNA repair protein *RAD51* in humans [11]. It is involved in the cellular response to DNA damage. It is essential for the repair of double-strand breaks (DSBs) through homologous recombination repair (HRR), specifically by facilitating strand invasion/exchange [12]. In normal cells, *RAD51* expression is precisely controlled, enhancing the accuracy of genome repair and preserving structural integrity. However, *RAD51* dysregulation is linked to tumor growth, metastasis, and resistance to therapy and has been shown in various human cancers, including BC [13]. Thus, *RAD51* exhibits predictive potential in malignancies and is expected to serve in the prognosis of therapeutic response and overall survival [14]. *RAD51* single-nucleotide polymorphisms (SNPs) are associated with increased susceptibility to BC, resistance to RT, and the emergence of new primary tumors [15]. Accordingly, this study aimed to investigate the levels and polymorphisms of *RAD51* in Iraqi women with BC before and after RT, along with its potential as a predictive biomarker for cancer risk and treatment response.

## Materials and Methods

### Subjects

Thirty newly diagnosed Iraqi BC females, aged 34–56 years, who attended Al-Amal National Hospital for Cancer Management between July 2024 and January 2025, were enrolled in this follow-up case-control study. Patients were selected and diagnosed by the consultant medical staff and a committee comprising a pathologist and oncologist, based on clinical examination, mammography, computed tomography (CT) scan, and histological findings. None of the patients received chemotherapy or RT at the first blood collection. Subsequently, the same thirty patients received RT and were followed up to complete their fractions for the second blood collection. The RT protocol administered was 3D-Conformal Radiation Therapy (CRT), including 40-50 Gy for 15-30 fractions (5 sessions/week). The exclusion criteria in this study include BC patients who received chemotherapy or RT, those with missed blood collection after RT, those suffering from other chronic diseases, those providing incomplete information, and pregnant or lactating women. As a control group, this study enrolled thirty age- and sex-matched healthy women with no history of medical illnesses.

### Sample collection

Regarding BC patients, blood samples were collected before the start of RT fractions. Thereafter, the patients were followed up, and additional blood samples were collected after the last fraction of RT. A total of 90 blood samples were collected from all participants and divided into two aliquots: whole blood for molecular and SNPs investigation, and serum for estimation of serum *RAD51* levels.

### Serum estimation of RAD51

A manufactured-on-request Sandwich ELISA kit (FineTest®, China) was used to quantitatively determine the serum levels of human *RAD51* protein in both patients and control groups [16].

### DNA extraction

Following the manufacturer’s instructions, the G-spin Total DNA Extraction Kit (INtRON Biotechnology, Korea) was used to extract DNA from EDTA blood samples. The recovered DNA was kept at −20 °C for subsequent polymerase chain reaction (PCR) amplification.

### DNA amplification

The template DNAs were amplified by PCR using specific primers for *RAD51*: forward primer 5’-TGGGAACTGCAACTCATCTGG-3’ and reverse primer 5’-GCGCTCCTCTCTCCAGCAG-3’ (IDT, USA). A total of 30 µl was used for the PCR amplification. To this, 1 µl of each primer (10 pmol) and 1.5 µl of DNA template were added to a tube containing 5 µl of PCR PreMix, followed by 21.5 µl of distilled water. The thermal cycling conditions of 37 cycles were performed using a Thermal Cycler (Labnet; USA) as following: 1 cycle of initial denaturation at 95ᵒC for 5 minutes; 35 cycles of denaturation (95ᵒC for 45 seconds), annealing (64ᵒC for 30 seconds), and extension (72ᵒC for 45 seconds); then one cycle of final extension for 5 minutes at 72ᵒC. After that, PCR products were visualized using 1.5% agarose gel electrophoresis and RedSafe stain.

### DNA sequencing and polymorphism analysis

The PCR products of *RAD51* were sequenced using the Sanger method performed by Macrogen Company, Korea, on an ABI-310 automated DNA sequencer (Applied Biosystems, USA). A homology search was conducted using the Basic Local Alignment Search Tool (BLAST) program, available online at the National Center for Biotechnology Information (NCBI) website (http://www.ncbi.nlm.nih.gov), and the BioEdit program. For SNP analysis, a multiple sequence alignment was performed using the Bio-ID program on the NCBI.

### Statistical analysis

Data were analyzed using SPSS software (version 27.0, USA) and presented as mean ± standard deviation (SD), with numbers and percentages provided when appropriate. A One-way ANOVA was conducted to compare the studied groups. Genotype and allele frequencies were computed based on the Hardy-Weinberg equilibrium (HWE) calculation, using Chi-square and Odds ratio for comparison and probability analysis. Differences were found to be significant at P<0.05.

## Results

### Serum RAD51 levels

**Figure 1:**
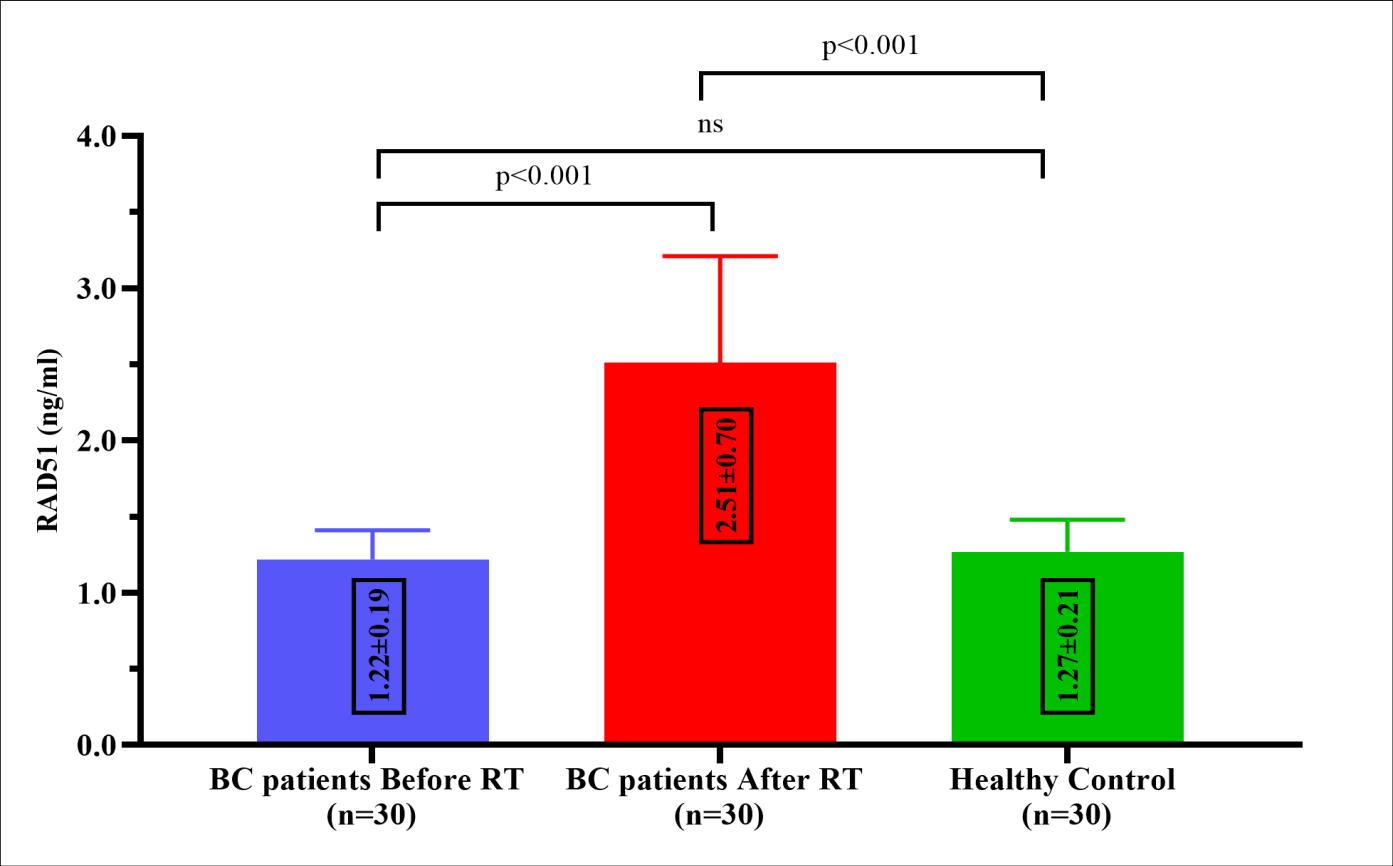
The Mean Levels of Serum *RAD51* Protein among the Studied Groups. BC, breast cancer; RT, radiotherapy.

Figure 1 illustrates that serum *RAD51* levels in BC patients after the final RT fraction were significantly higher than both pre-treatment levels and those of healthy controls (2.51 ± 0.70 vs. 1.22 ± 0.19 vs. 1.27 ± 0.21 ng/ml, P<0.001). Before initiating RT fractions, BC patients demonstrated reduced *RAD51* levels compared to healthy controls, with no significant difference (1.22 ± 0.19 vs. 1.27 ± 0.21 ng/ml, P > 0.05).

### RAD51 gene amplification

**Figure 2:**
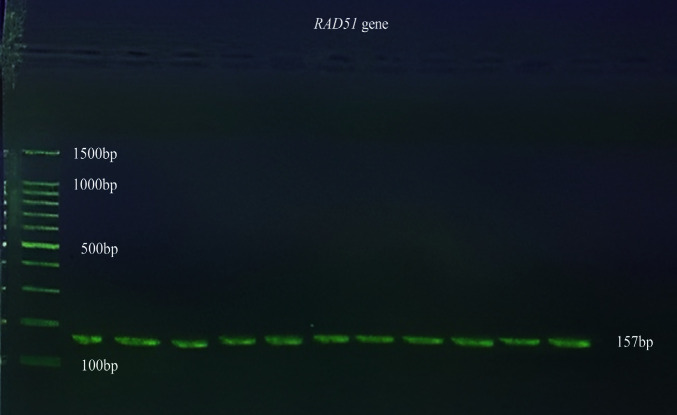
Gel Electrophoresis of PCR Products of the* RAD51 *Gene of Exon 1 on 1.5% Agarose at 5 volts/cm² for 90 Minutes.

We applied PCR amplification to identify the *RAD51* gene in exon 1, followed by sequencing and SNP analysis. Figure 2 displays that the *RAD51* gene’s exon 1 produced a PCR product of approximately 157 bp, which was electrophoresed on an agarose gel and visualized under UV light.

### Polymorphisms of the RAD51 gene

**Figure 3:**
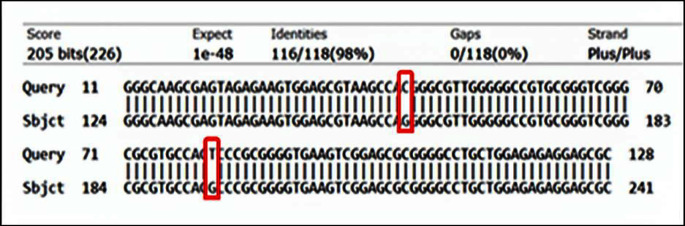
Sequence Alignment of Exon 1 in the *RAD51* Gene among the Studied Groups

Two SNPs, rs1801320 (G>C) and rs1801321 (G>T), were identified through sequencing alignment of exon 1 in the *RAD51* gene, as demonstrated in Figure 3. The prevalence of these SNPs among BC patients was 20% and 70%, respectively, compared to 10% and 20% in healthy controls.

### Genotype and allele frequency of RAD51 SNPs

In Table 1, 26.7% of BC patients had the mutant (GT) genotype before RT, compared to 20.0% after RT. Workers and healthy controls had a 3.3% frequency for this genotype. Results suggest a substantial risk factor (P < 0.001; OR = 11.07, 95% CI = 3.20–37.87). BC patients before RT (13.3%), post-RT (10.0%), and controls (3.3%) have the mutant allele (T). Pattern suggests a substantial risk factor (P < 0.00; OR = 4.92, 95% CI = 1.82-13.35). Although BC patients showed a higher risk for the mutant (GC) genotype compared to controls (P > 0.05; OR = 2.25, 95% CI = 0.50-9.93) and for the mutant (C) allele (P > 0.05; OR = 2.11, 95% CI = 0.50-8.87), there were no statistically significant differences between the groups studied, as shown in Table 2. 

## Discussion

Considering the rising prevalence of BC globally and the potential for RT resistance to develop in patients, it is imperative to assess the efficacy of RT before completing all its sessions. Locally, to our knowledge, this study is the first to estimate serum levels of *RAD51* protein in BC patients both before and after RT. The results showed significantly increased levels in patients following RT compared to pre-treatment levels and those of healthy controls. These findings are consistent with studies indicating that *RAD51* expression increased after RT in BC patients [17, 18]. This expression demonstrated a positive correlation with cancer progression and metastasis, suggesting its potential as a significant clinical marker for cancer prognosis and a therapeutic target. Other studies indicated a reduced *RAD51* expression compared to pre-treatment, along with downregulation of HRR and improved BC outcomes, suggesting that elevated *RAD51* is associated with tumor formation and poor prognosis [19, 20]. The role of *RAD51* as a DNA repair protein, essential for the repair of DSBs, correlates with its increased levels. Hence, its elevated expression represents a typical cellular response to RT-induced DNA damage [12]. 

Regarding the PCR amplification of exon 1 of the *RAD51* gene, it was utilized, revealing a product size of 157 bp, which is responsible for gene regulation. This finding is consistent with studies that reported the same product size of the *RAD51* gene [21, 22]. DNA sequencing analysis revealed two SNPs in the 5’ untranslated region (5’UTR) of exon 1 of the *RAD51* gene: rs1801320 (G>C) and rs1801321 (G>T). These polymorphisms are located in the regulatory region of the *RAD51* promoter and have been proposed to be associated with mRNA stability and expression, thereby hindering apoptosis and promoting cell survival [22]. This study found that BC patients with the rs1801321 SNP had a higher rate of the GT genotype and T allele, which were significant risk factors compared to healthy individuals. This variation was significantly reduced in BC patients after RT, compared to patients before treatment, indicating that RT may restore the gene to its wild-type state through RT-induced DNA repair mechanisms. For Iraqi individuals, the SNP *RAD51* rs1801321 (G>T) has been newly registered in the NCBI database with the accession number PV661828. With limited published studies on this topic, the findings are consistent with research conducted in Saudi Arabia [23] and India [24], which showed a significant association between the *RAD51* variant (172G>T) and BC risk. Additionally, the observed cytoplasmic expression in cancer cells is linked to poorer prognostic outcomes. Moreover, a meta-analysis study indicated that the *RAD51* G172T polymorphism is linked to a heightened risk of BC, particularly within the Arab population [25]. This SNP is located in the transcription factor binding site, resulting in enhanced activity of the *RAD51* promoter and an increased capacity for DSB repair pathways. In a similar context, studies indicate that BC patients with the *RAD51* GT/TT genotype show a higher incidence of radiation-induced adverse events, including dermal, cardiac, or lung toxicity in patients undergoing RT [26, 27]. 

The *RAD51* rs1801320 (G>C) polymorphism disclosed a higher frequency of the GC genotype and C allele in BC patients, showing a 2-fold risk compared to healthy controls. However, no statistically significant differences were observed between BC patients before and after RT, and the control group. This finding indicates that the G>C substitution elevates the risk of developing BC in Iraqi women, in contrast to those with the GG genotype, who are protected. The *RAD51* rs1801320 (G>C) SNP has been newly registered in the NCBI database under the accession number PV661827 for Iraqi BC patients prior to RT. Our results aligned with research conducted in Turkey, Iraq, the Philippines, and Italy, which found that there was a lack of association between the heterozygous (GC) genotype of the *RAD51* (G135C) polymorphism and BC risk, despite the increased prevalence of the GC genotype and C allele in BC patients compared to healthy controls [28-31]. However, several studies performed in Saudi Arabia, Jordan, Poland, and Pakistan have shown that the mutant genotype/allele (CC/C) of the *RAD51* rs1801320 is strongly associated with an increased risk of BC and worse prognostic outcomes [32-35]. This finding suggests the potential use of this SNP for cancer risk assessment and therapeutic strategies targeting DNA repair mechanisms. As mentioned earlier, the G>C polymorphism in the 5’ UTR of *RAD51* increases its promoter activity, resulting in *RAD51* overexpression, unregulated HRR, and genomic instability, which are linked to resistance to RT [13]. Nevertheless, BC patients with the *RAD51* rs1801320 (G>C) SNP showed no association with radiation-related adverse effects in normal tissues after RT, according to several published studies [15, 26, 27]. Otherwise, a study conducted in India revealed that the GC/CC genotype of *RAD51* rs1801320 was strongly correlated with a lowered risk of BC [36]. Inconsistencies in results could arise due to differences among populations, treatment protocols, or different follow-up periods. The study’s limitation is its restricted sample size, which complicates the identification of small to moderate associations between *RAD51* polymorphism and BC risk. Therefore, the estimates provided must be interpreted cautiously and may not be generalizable to the broader population. Consequently, larger and more rigorously designed studies are required to validate these findings.

In conclusion, this study related the *RAD51* (G>T) polymorphism to a higher risk of BC in the Iraqi population. Additionally, patients with the GT genotype / T allele benefited significantly from RT, suggesting that *RAD51* might be exploited as a predictive biomarker for cancer risk and RT outcomes. Since no relation was found between BC risk and the *RAD51* (G>C) polymorphism, the GG genotype may have a protective effect on BC in Iraqi women. Future larger studies are necessary to investigate DNA repair genetic variations in BC susceptibility.

**Table 1:** Genotype and Allele Frequency of *RAD51* Polymorphisms (rs1801321) among the Studied Groups.

Genotype and allele frequency		BC patients		Healthy Controls (n=30)	Total	OR (95%CI)	P-value
		Before RT (n=30)	After RT (n=30)				
SNP rs1801321 (G>T)	GG	6	12	24	42	1.00 Ref.	
		6.7%	13.3%	26.7%	46.7%		
	GT	24	18	6	48	11.07 (3.20-37.87)	<0.001
		26.7%	20.0%	6.7%	53.3%		
	TT	0	0	0	0	NA	NA
		0.0%	0.0%	0.0%	0.0%		
	G	36	42	54	132	1.00 Ref.	
		20.0%	23.3%	30.0%	73.3%		
	T	24	18	6	48	4.92 (1.82-13.35)	<0.001
		13.3%	10.0%	3.3%	26.7%		

SNP, single nucleotide polymorphism; BC, breast cancer; RT, radiotherapy; OR, Odds ratio; CI, Confidence Interval; NA, not applicable.

**Table 2:** Genotype and Allele Frequency of *RAD51* Polymorphisms (rs1801320) among the Studied Groups

Genotype and allele frequency		BC patients		Healthy Controls (n=30)	Total	OR (95%CI)	P-value
		Before RT (n=30)	After RT (n=30)				
SNP rs1801320 (G>C)	GG	24	24	27	75	1.00 Ref.	
		26.7%	26.7%	30.0%	83.3%		
	GC	6	6	3	15	2.25 (0.50-9.93)	0.286
		6.7%	6.7%	3.3%	16.7%		
	CC	0	0	0	0	NA	NA
		0.0%	0.0%	0.0%	0.0%		
	G	54	54	57	165	1.00 Ref.	
		30.0%	30.0%	31.7%	91.7%		
	C	6	6	3	15	2.11 (0.50-8.87)	0.307
		3.3%	3.3%	1.7%	8.3%		

SNP, single nucleotide polymorphism; BC, breast cancer; RT, radiotherapy; OR, Odds ratio; CI, Confidence Interval; NA, not applicable.

## Author Contribution Statement

Haael Subhi Abbas and Ali Abdul Hussein Mahdi conceptualized and designed the study, as well as conducted the tests. Nihad Khalawe Tektook executed the sample collection and performed the statistical analysis. Haael Subhi Abbas, Ali Abdul Hussein, and Nihad Khalawe Tektook participated in the execution of the research, the analysis of the results, and the drafting of the manuscript.
